# Screening Diabetic Retinopathy Using an Automated Retinal Image Analysis System in Independent and Assistive Use Cases in Mexico: Randomized Controlled Trial

**DOI:** 10.2196/25290

**Published:** 2021-08-26

**Authors:** Alejandro Noriega, Daniela Meizner, Dalia Camacho, Jennifer Enciso, Hugo Quiroz-Mercado, Virgilio Morales-Canton, Abdullah Almaatouq, Alex Pentland

**Affiliations:** 1 MIT Media Laboratory Massachusetts Institute of Technology Cambridge, MA United States; 2 Prosperia Salud Mexico City Mexico; 3 Retina Department Asociación para Evitar la Ceguera en México Mexico City Mexico; 4 Engineering Academic Division Instituto Tecnológico Autónomo de México Mexico City Mexico; 5 Posgrado de Ciencias Bioquímicas Universidad Nacional Autónoma de México Mexico City Mexico; 6 Sloan School of Management Massachusetts Institute of Technology Cambridge, MA United States

**Keywords:** diabetic retinopathy, automated diagnosis, retina, fundus image analysis

## Abstract

**Background:**

The automated screening of patients at risk of developing diabetic retinopathy represents an opportunity to improve their midterm outcome and lower the public expenditure associated with direct and indirect costs of common sight-threatening complications of diabetes.

**Objective:**

This study aimed to develop and evaluate the performance of an automated deep learning–based system to classify retinal fundus images as referable and nonreferable diabetic retinopathy cases, from international and Mexican patients. In particular, we aimed to evaluate the performance of the automated retina image analysis (ARIA) system under an independent scheme (ie, only ARIA screening) and 2 assistive schemes (ie, hybrid ARIA plus ophthalmologist screening), using a web-based platform for remote image analysis to determine and compare the sensibility and specificity of the 3 schemes.

**Methods:**

A randomized controlled experiment was performed where 17 ophthalmologists were asked to classify a series of retinal fundus images under 3 different conditions. The conditions were to (1) screen the fundus image by themselves (solo); (2) screen the fundus image after exposure to the retina image classification of the ARIA system (ARIA answer); and (3) screen the fundus image after exposure to the classification of the ARIA system, as well as its level of confidence and an attention map highlighting the most important areas of interest in the image according to the ARIA system (ARIA explanation). The ophthalmologists’ classification in each condition and the result from the ARIA system were compared against a gold standard generated by consulting and aggregating the opinion of 3 retina specialists for each fundus image.

**Results:**

The ARIA system was able to classify referable vs nonreferable cases with an area under the receiver operating characteristic curve of 98%, a sensitivity of 95.1%, and a specificity of 91.5% for international patient cases. There was an area under the receiver operating characteristic curve of 98.3%, a sensitivity of 95.2%, and a specificity of 90% for Mexican patient cases. The ARIA system performance was more successful than the average performance of the 17 ophthalmologists enrolled in the study. Additionally, the results suggest that the ARIA system can be useful as an assistive tool, as sensitivity was significantly higher in the experimental condition where ophthalmologists were exposed to the ARIA system’s answer prior to their own classification (93.3%), compared with the sensitivity of the condition where participants assessed the images independently (87.3%; *P*=.05).

**Conclusions:**

These results demonstrate that both independent and assistive use cases of the ARIA system present, for Latin American countries such as Mexico, a substantial opportunity toward expanding the monitoring capacity for the early detection of diabetes-related blindness.

## Introduction

### Impact of Diabetes

Diabetes is one of the most challenging health problems in the world, affecting more than 400 million people. Particularly, diabetes threatens the health care systems of low- and middle-income countries, where 80% of the world’s diabetic population live [1,2]⁠. Diabetes is a multifactorial and complex disease with a strong genetic component. In this regard, it has been demonstrated that Hispanic/Latino people have a greater susceptibility to develop type II diabetes, as well as diabetes-associated complications, including renal insufficiency and visual impairment [1-4].

In 2015, there were more than 41 million adults diagnosed with diabetes in Latin America and Caribbean countries, making it one of the major causes of premature death and disability in the region [5,6]⁠. Particularly, Mexico ranked sixth among the world’s diabetes prevalence in 2015 and second among Latin America, only after Brazil [7,8]. It is estimated that 26 million adults live in Mexico with diabetes or prediabetes, and only half of them have been diagnosed. Diabetes and its related complications are the first cause of disability and the third cause of death in the country, largely impacting productivity, life quality, and the economy [5].

### Evolution and Treatment of Diabetic Retinopathy

Diabetic retinopathy (DR) is the most common complication in advanced or uncontrolled diabetic patients and is the leading cause of irreversible vision loss in working-age adults [9,10]. DR is a microvascular complication that emerges in diabetic patients as a consequence of chronic hyperglycemia that contributes to blood vessel damage in the retina, causing a combination of fluid leakage, swelling of the surrounding tissue, blood flow obstruction, and abnormal neovascularization [9,10].

DR progression is slow, gradual, and reversible in its first stage. However, if not treated promptly, it can lead to irreversible blindness⁠. According to the International Clinical Diabetic Retinopathy Severity Scale, the first stage of DR is classified as mild nonproliferative diabetic retinopathy (NPDR), which is characterized by the presence of at least 1 microaneurysm and is highly reversible through blood pressure, cholesterol, and sugar level control. Only very rare cases that present macular edema (swelling of fluid and protein deposits on or under the macula) might require laser photocoagulation or intravitreal injections. Without adequate diabetic control, the disease advances to moderate and severe NPDR stages, which include the presence of hemorrhages, microaneurysms, hard exudates, venous beading, or intraretinal microvascular abnormalities. At these stages, metabolic control is not sufficient to stop the disease progression, and the patient will require invasive treatments such as photocoagulation and intravitreal antivascular endothelial growth factor agents or corticosteroids. The most advanced stage is proliferative DR and is characterized by neovascularization, preretinal hemorrhages, hemorrhages in the vitreous, traction retinal detachments, or macular edema. Proliferative DR is treated with the more aggressive laser therapy called scatter or pan-retinal photocoagulation; intravitreal injection; and, in some cases, vitreoretinal surgery, which removes scar tissue or blood from the vitreous cavity to repair retinal detachments or treat macular holes [10-13].

To increase early detection and prevent the progression of DR to advanced stages, diabetic patients are recommended to have annual or semiannual retinal screenings beginning at the moment when they are diagnosed with diabetes. However, according to data from the Diabetic Retinopathy Barometer, 27% of people living with diabetes declared that they never discussed eye complications with their doctors before the onset of complications, and only 13% of the diabetic population have visited an ophthalmologist after their diagnosis [4,14]. Through frequent, preventive screenings, 70% of the cases can be captured at the initial stages of the disease and treated with noninvasive strategies such as metabolic control or photocoagulation [15]. Unfortunately, in most developing countries, there is no ophthalmological attention at primary care clinics, and it is only when diabetic patients develop vision attenuation that they are referred to second- and third-level hospitals to be screened, diagnosed, and treated [16]. At this point, significant retinal damage has occurred, and, even with invasive vitreoretinal surgery or photocoagulation, vision cannot be restored.

The limited access to ophthalmologists and retina specialists at primary care clinics, due to financial and staff limitations at national health care institutions, precludes the continuous monitoring of diabetic patients in low- and middle-income countries such as Mexico.

### Challenges of Diabetic Retinopathy Screening on a Large Scale

In Mexico, DR is a leading cause of irreversible blindness among the working-age population [4,13]⁠. Approximately 30% of the patients diagnosed with diabetes develop DR, and, based on the predictions of diabetes increasing in prevalence, by 2045, there will be 245 million people with DR lesions and 77 million people with vision-threatening DR [17].

One of the main limitations for the establishment of a systematic eye-screening program is the limited availability of ophthalmologists and their unequal distribution around the country. Based on the 2013 registry of society-affiliated ophthalmologists from the Mexican Society of Ophthalmology, the average number of ophthalmologists per 100,000 people is lower (2.68 per 100,000) than the average among Latin American countries (5.27 per 100,000). There is a particularly worrying distribution in rural areas, with 2 ophthalmologists per 100,000 people [18].

In particular, in low- and middle-income countries such as Costa Rica, Peru, and India, there have been several efforts to implement DR screening programs targeting the limitation of ophthalmologists with mobile screening units integrated with telemedicine [19-21]. In these contexts, 2 key factors were identified for achieving cost-effectiveness of these strategies: (1) accurate identification of the risk population and (2) optimization of the number of people screened per unit of time [21]. Notably, these 2 factors can be improved by leveraging automated retinal image analysis (ARIA) systems such as the one in this study.

### ARIA for Diabetic Retinopathy Screening

In recent years, the combination of the development of advanced statistical methods, the greater availability of data, and the substantial increase in computing power has allowed for the application of advanced computational methodologies, including artificial intelligence (AI), in diverse social and medical domains. Among the use of AI for social welfare, AI applications in health care domains are one of the fastest growing sectors, with a compound annual growth rate above 40% during the period between 2014 and 2021 [22]. AI tools have been successfully applied to diagnostics, therapeutics, population health management, administration, and regulation, showing a capacity to augment societies’ access to health care and improve the coverage and quality of the services provided.

Ultimately, AI applications in health care present opportunities to improve overall quality of life, patients’ prognoses, and optimization of human and financial resources [23]. In particular, ARIA systems have emerged as a promising solution to increase early detection of DR at primary care clinics, particularly, in resource-constrained developing countries, thereby improving health outcomes, avoiding incapacitating complications, and reducing treatment costs.

ARIA systems analyze retinal fundus images by applying techniques such as deep learning (DL) to classify diabetic patients in (1) cases without retinal lesions associated to DR (nonreferable output) and (2) cases that need to undergo examination by an ophthalmologist to confirm diagnosis and define treatment (referable output) [24-28]. As of today, various analysis systems have been developed and implemented on the market in European countries, Canada, and the United States. However, very few have been tested in Latin America and Caribbean countries to evaluate their performance and usability in the particular resource-constrained settings of these countries [29]. To determine qualities of successful implementation in these countries, research must investigate patients’ ethnicities, the training of health care personnel, community openness to new technologies, and hospital resources.

### Aims and Key Findings of the Study

This study aimed to evaluate the performance of a DL-based ARIA system that classifies retinal fundus images in nonreferable or referable circumstances, based on the presence of DR damage, as well as the potential benefits of its use as an assistive tool for ophthalmic doctors. We also completed a randomized controlled trial where the performance of the ARIA system was compared with the accuracy of 17 ophthalmologists from one of the most reputable ophthalmic hospitals in Mexico, Hospital de la Ceguera, which is part of the “Association to Avoid Blindness in Mexico” (APEC). In particular, the performances of ophthalmologists in 3 experimental conditions were assessed: 1 independent condition, in which the ophthalmologists assessed the images independently from the ARIA system, and 2 assistive conditions, in which either ophthalmologists observed and were influenced by the ARIA system’s classification and confidence or an ARIA system–generated, attention heatmap highlighted probable DR lesions in the retina.

The key findings were that the ARIA system developed using a DL strategy was able to classify referable vs nonreferable cases with an area under the receiver operating characteristic curve (AUROC) of 98%, a sensitivity of 95.1%, and a specificity of 91.5% for international patient cases. There was an AUROC of 98.3%, a sensitivity of 95.2%, and a specificity of 90% for Mexican patient cases. For Mexican patient cases, the ARIA system performance was more successful than the average performance of the 17 ophthalmologist participants in the study. Moreover, we found that the ARIA system can be useful as an assistive tool, as we found significant improvement in the specificity in the experimental condition where participants were able to consider the answer of the ARIA system as a second opinion (87.3%)*,* compared with the specificity of the condition where participants assessed the images independently (93.3%; *P*=.05).

Hence, this study aimed to demonstrate the high potential value of the use of ARIA systems, in both independent and assistive schemes, toward the goal of effective mass screening for the early detection of DR in developing countries such as Mexico.

## Methods

### ARIA System

#### ARIA System Design

**Figure 1:**
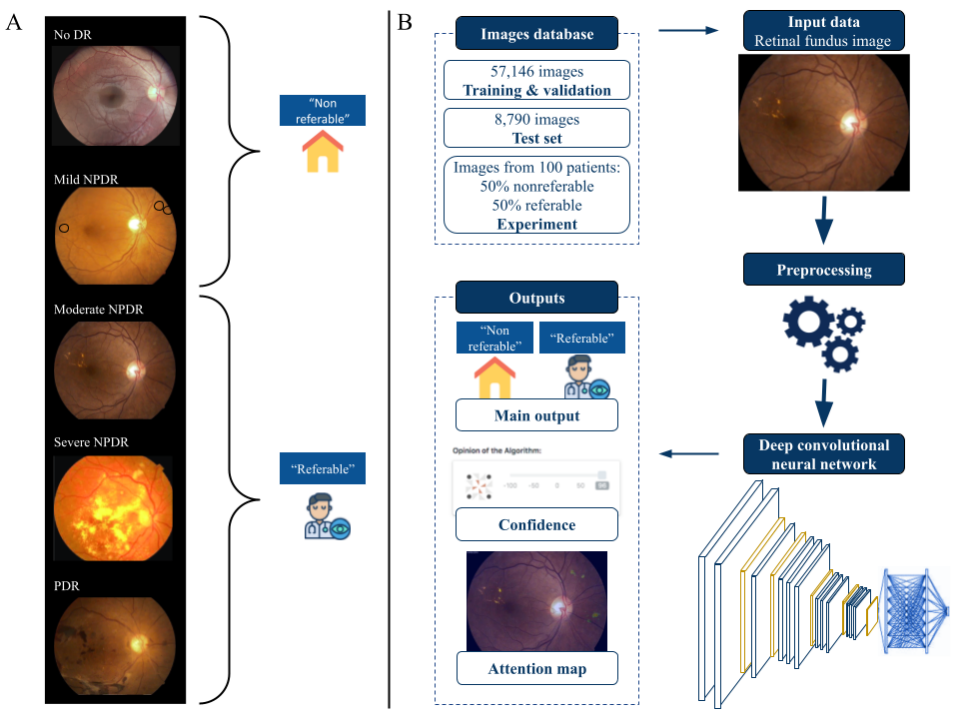
Deep learning–based automated retinal image analysis system. (A) Example of classified retinal fundus images according to the International Clinical Diabetic Retinopathy Severity Scale used for the training data. (B) Flow chart describing the design of the automated retinal image analysis system; the data used for training, validation, and testing; and the algorithm’s outputs. DR: diabetic retinopathy; NPDR: nonproliferative diabetic retinopathy; PDR: proliferative diabetic retinopathy.

The ARIA system consists of an image preprocessing module and an image analysis module that returns a binary referable and nonreferable DR classification; the level of confidence of that classification; and an attention map that shows, pixel-wise, the indicative features for referable DR according to the model (Figure 1). The models constituting the ARIA system were implemented using the Keras library with the Tensorflow backend [30] in Python 3.5 [31].

Images from all datasets were annotated by ophthalmic specialists for 5-class identification according to the International Clinical Diabetic Retinopathy Severity Scales (ICDRSS) and subsequently labeled as nonreferable or referable DR [32]. Table 1 describes the classification, and Figure 1A provides a graphical example. The gold standard classification used for the experimental phase of the study was provided by 3 retina specialists, as described in the following subsections.

**Table 1:** International Clinical Diabetic Retinopathy Severity Scale and its classification for the automated retinal image analysis system [32].

ARIA^a^ system classification	DR^b^ severity scale	Ophthalmoscopy findings
Nonreferable	No apparent retinopathy (no DR)	No abnormalities
	Mild nonproliferative DR (mild DR)	Microaneurysms only
Referable	Moderate nonproliferative DR (moderate DR)	More than just microaneurysms but less than severe nonproliferative diabetic retinopathy
	Severe nonproliferative DR (severe DR)	≥20 intraretinal hemorrhages in each of 4 quadrants, definite venous beading in 2 quadrants, or prominent intraretinal microvascular abnormalities in 1 quadrant. No signs of proliferative retinopathy.
	Proliferative DR	Neovascularization or vitreous/preretinal hemorrhage.

^a^ARIA: automated retinal image analysis. ^b^DR: diabetic retinopathy.

#### Preprocessing

Before classifying the images and training the algorithms, a preprocessing procedure was applied. The procedure consisted of cropping the background to eliminate noninformative areas, padding the image to guarantee consistent squared image ratios, resizing the image to 224×224 pixels, and normalizing pixel values to the range 0-1.

#### Image Classification Model

The model used for image classification consisted of a deep convolutional neural network [33,34]. The network architecture developed for this project consisted of 16 convolutional layers, a dense layer of 1024 neurons, 2 dropout layers to avoid overfitting, and a binary classification layer of a single unit with sigmoid activation. This architecture took the VGG model published by Simonyan and Zisserman [34] as a starting point. Hence, the model output is a value between 0 and 1, which may be interpreted as the confidence of the model regarding a referable DR classification. Lastly, a threshold of 0.5 was used to classify nonreferable (<0.5) and referable (≥ 0.5) DR.

The model was trained on an international dataset, of which most images were taken in primary care clinics in California, United States [35]. The training subset had 57,146 images (16,458/57,146, 28.80% with referable DR; 45,602/57,146, 79.80% gradable), and the evaluation subset had 8,790 images (694/8790, 7.90% with referable DR; 7067/8790, 80.40% gradable). The training and test subsets followed the same distribution used by Voets and colleagues [36]. Considering real-life scenarios, the training and validation datasets included images from different types of cameras and of different qualities (ie, with artifacts, out of focus, underexposed, or overexposed).

#### Attention Heatmaps

**Figure 2:**
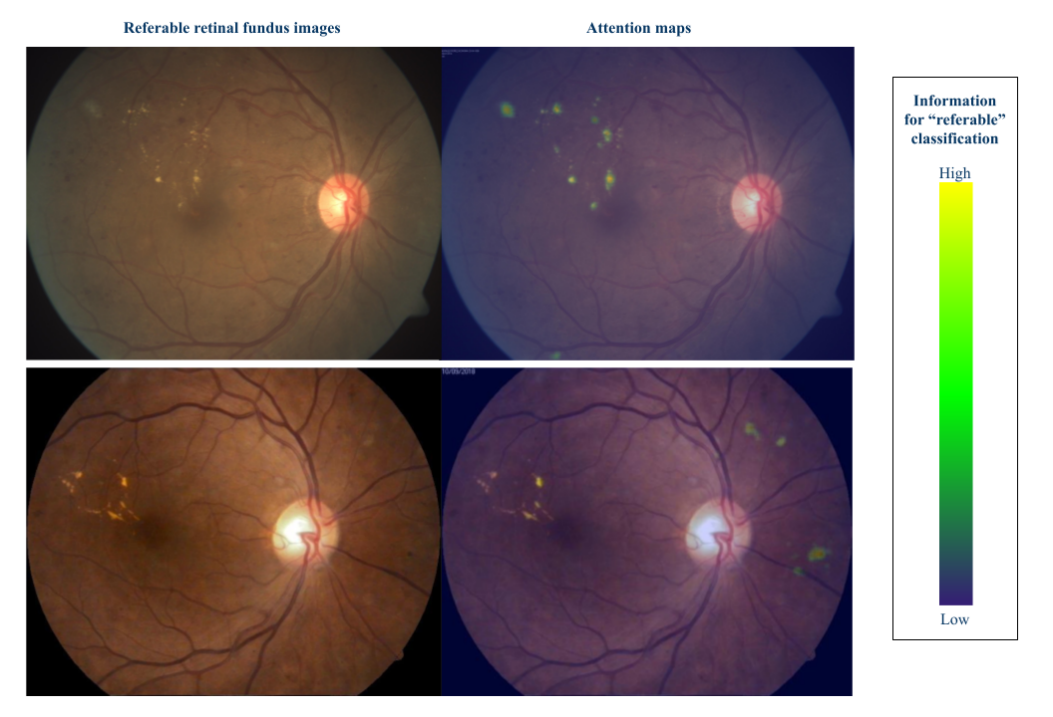
Attention heatmaps for 2 referable images. Green and yellow colors indicate regions in the image that provide information to the algorithm to classify the image as referable.

Attention heatmaps were developed to show lesion areas in the image by highlighting each pixel according to their importance to a referable DR classification, according to the model. These heatmaps were obtained by applying one of the most effective methods for building saliency maps on images, the layer-wise relevance propagation method, with an alpha-beta rule [37,38]. In essence, the layer-wise relevance propagation method redistributed the output value throughout the layers until the input layer (input image) was reached. Figure 2 shows examples of fundus images and the heatmaps generated using the methodology described.

### Study Populations

We had 17 ophthalmologists from the Mexican ophthalmic hospital participating in the experimental study, and 3 retina specialists from the same institution participated in the generation of the gold standard. The 17 ophthalmologists evaluated 45° macula-centered fundus images from 100 Mexican patients, where 50% (50/100) had nonreferable DR and 50% (50/100) had referable DR levels. Each ophthalmologist evaluated 45 retinal images, in order for each image to be evaluated more than once. The ophthalmologists were retina specialization resident students, where 3 residents were in their second year, 12 were in their third year, and 2 were in their fourth year of residency.

### Experimental Design

#### Overview of Study Design

**Figure 3:**
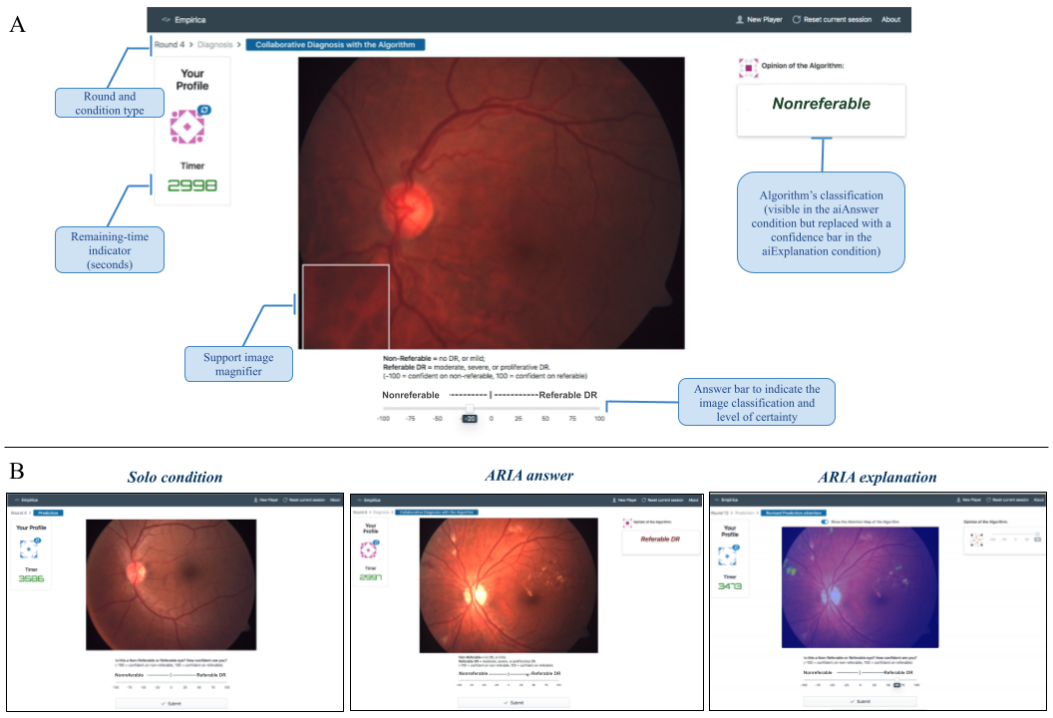
Web-platform design for patient-case classification. (A) Visual indicators and components of the classification window. (B) Visualization of the 3 experimental conditions. ARIA: automated retinal image analysis; DR: diabetic retinopathy.

We conducted a randomized controlled experiment to assess the performance of the ARIA system in comparison with ophthalmic doctors from the Mexican ophthalmic hospital and to evaluate the potential benefits of using the system as an assistive tool for doctors. To achieve this, a web-based experiment platform was developed where ophthalmologists evaluated fundus retinal images under 3 different conditions—solo, ARIA answer, and ARIA explanation*—*described below. The platform was developed based on the Empirica framework [39]. Figure 3 displays the main screens of the web platform used in this experiment.

#### Gold Standard and Image Quality

To generate a gold standard, the fundus images of all patient cases used in the experiment were graded by 3 retina specialists of the ophthalmic hospital, and a majority rule was used (ie, if there was a disagreement in the nonreferable/referable label, the label selected by 2 of 3 experts was considered the gold standard). We used the same web-based platform described in Figure 3 for image grading. The retina specialists also graded the image quality, and images graded as bad quality were not considered for the experiment. From the remaining images, 50 images from patients with referable DR and 50 images from patients with nonreferable DR were selected at random to be used for the study. According to the ICDRSS, the selected images had the following distribution: 49 with no apparent retinopathy, 1 with mild DR, 33 with moderate DR, 12 with severe nonproliferative DR, and 5 with proliferative DR. Since these images were taken at an ophthalmic hospital, most patients with DR were under treatment and therefore had more advanced DR stages (moderate, severe, and proliferative DR).

#### Experimental Conditions

The experiment followed a within-subjects design, where each ophthalmologist evaluated 45 randomly selected fundus images (from 45 different patients), 15 for each of the 3 treatment conditions: solo, ARIA answer, and ARIA explanation. The ophthalmologists were first asked to evaluate 15 fundus retinal images in the solo condition, followed by 30 images that randomly alternated between the ARIA answer and the ARIA explanation conditions. The 15 images in each condition subset were randomly selected for each participant without replacement from all images available for the experiment, generating a rough balance in the proportion of referable and nonreferable images across conditions. In particular, the average proportion of referable images was 49.8% (127/255) for the solo condition, 52.5% (134/255) for the ARIA answer condition*,* and 46.7% (119/255) for the ARIA explanation condition. In addition, Multimedia Appendix 1 reports the average number of observations of each ICDRSS class for each treatment condition.

In the solo condition, participants responded to the task in isolation, without any exposure to the ARIA system. In contrast, in the ARIA answer condition, participants were exposed to the binary answer of the ARIA system (ie, nonreferable or referable), as a second opinion, and then asked to submit their postexposure answer. The ARIA explanation condition was identical to the ARIA answer condition, with the exception that participants were shown not only the binary answer of the ARIA system but also its level of confidence and attention heatmap.

Finally, after completing all the classification tasks, the ophthalmologists were asked to submit an optional feedback survey about their experience.

The study was reviewed and approved by the Committee on the Use of Humans as Experimental Subjects at the Massachusetts Institute of Technology, and all participants provided explicit consent prior to their participation.

## Results

### ARIA’s Independent Performance

**Figure 4:**
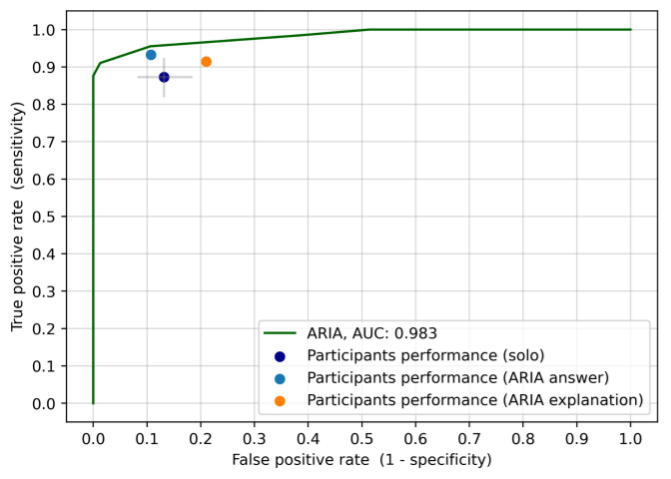
Receiver operating characteristic curve of the ARIA system compared with the ophthalmologist’s accuracy under the 3 experimental conditions (solo, ARIA answer, and ARIA explanation). Grey lines indicate 95% CIs for the solo condition. ARIA: automated retinal image analysis; AUC: area under the curve.

The ARIA system was first tested in a large dataset of international cases. It achieved an out-of-sample area under the receiver operating characteristic curve (AUROC) of 98% (Multimedia Appendix 1). In particular, using a given acceptance threshold, the ARIA system achieved a sensitivity of 95.1% and a specificity of 91.5%. Most importantly, the ARIA system also displayed high accuracy classifying images from patients from the Mexican ophthalmic hospital, where it had an AUROC of 98.3%, a sensitivity of 95.2%, and specificity of 90% (Figure 4).

### ARIA’s Assistive Performance

Figure 4 shows the sensitivity and false positive rate (false positive rate = 1 – specificity) for each condition—solo, ARIA answer, and ARIA explanation—and compares them with the receiver operating characteristic curve of the ARIA system. The average sensitivity in the solo condition across the 17 participants was 87.3%, and the average specificity was 86.8%. In comparison, the average sensitivity and specificity across the 17 participants for the ARIA answer condition were 93.3% and 89.3%, respectively, and the average sensitivity and specificity across participants for the ARIA explanation condition were 91.5% and 79%, respectively.

The joint analysis of the ARIA system performance for Mexican patients, compared with the 3 experimental conditions involving ophthalmologist assessments, showed that the ARIA system is more accurate than the average accuracy of participants under any of the exposure conditions. In particular, the ARIA system increased sensitivity from 87.3% to 93.3% (*P*=.05; vertical movement between the dark blue dot and the green line in Figure 4) while maintaining participants’ specificity at 86.8%. Compared with the solo condition, the ARIA system also increased specificity to 100% while maintaining participants’ average sensitivity at 87.3% (horizontal movement from the dark blue dot leftwards to the green line in Figure 4).

Most interestingly, Figure 4 shows that exposure to the ARIA system was able to improve the performance of human experts, particularly, in the ARIA answer condition, which significantly improved the sensitivity and specificity compared with the solo condition (distance between dark blue and light blue dots in Figure 4). However, performance in the ARIA explanation condition had mixed results, showing improved sensitivity but worse specificity (distance between dark blue and orange dots in Figure 4).

**Figure 5:**
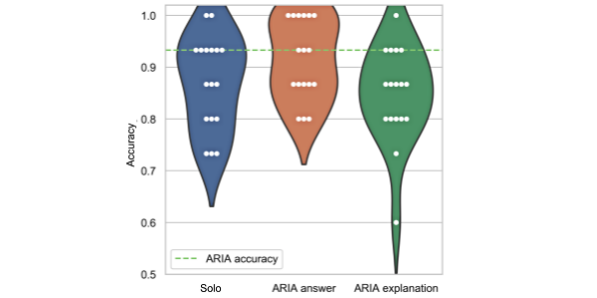
Influence of the ARIA system on the ophthalmologists’ decisions: ophthalmologists’ performance after exposure to the ARIA answer or the ARIA explanation condition outputs. ARIA: automated retinal image analysis.

Figure 5 provides more detail on the effect that exposure to information of the ARIA system had on the performance of ophthalmologists. In particular, it shows that the accuracy (% of correct answers) of the 17 experts consistently improved in the ARIA answer condition, shifting the distribution upwards and decreasing the variance across participants. For example, while only 2 participants had a perfect score in the solo condition, up to 6 participants had a perfect score in the ARIA answer condition. However, the ARIA explanation condition had mixed beneficial and detrimental effects on participants’ accuracy and increased the variance of performance across participants compared with the solo condition.

## Discussion

### Principal Findings

The number of people living with diabetes by 2045 is projected to reach 700 million people worldwide [7,40]. This means that routine eye screening might prevent vision loss in approximately 230 million patients. Just in Mexico, the prevention of DR would implicate savings of up to US $10 million for the 3 main public health care institutions [41]. The development of ARIA systems represents a possible solution to the increasing demand of eye screenings in health care systems, particularly, in limited-resource settings. However, it has been shown that acceptance of the human factors involved in the field processes are critical for the effective implementation of screening systems [42,43].

In this study, we successfully developed and evaluated a DL-based ARIA system to determine its performance as an independent decision-making system, as well as a supportive tool for health care professionals. As an independent decision-making tool, the ARIA system outperformed the average ophthalmologist participant in the experiment. On the other hand, as a supportive tool, the ARIA system exerted a strong influence on the opinion of human participants. However, its effect depended on the output’s format, highlighting the importance of a well-designed platform that has been user-tested with final users.

### ARIA’s Independent Performance

The DL-based ARIA system presented in this work was evaluated with a subset of retinal images from international patient cases and an image set of patients from a Mexican ophthalmic hospital. In both datasets, the ARIA system outperformed the average sensitivity and specificity of 17 ophthalmology residents of retina specialty.

The sensitivities (95.1% and 95.2% for the international and Mexican datasets, respectively) are comparable to those reported for 7 other automated DR screening systems assessed in a systematic review, whose sensitivity values were between 87% and 95% [44]. On the other hand, the specificities reached by our ARIA system (91.5% and 90% for the international and Mexican datasets, respectively) were higher than the average specificity values of between 49% and 69% reported by Nørgaard and Grauslund [44]. Also, our system’s sensitivity and specificity were comparable with those reported for commercial DR screening technologies with DL features, whose sensitivity and specificity values were 85%-99.3% and 68.8%-97.9%, respectively [45]. Compared with these commercial DR screening technologies, our ARIA system has one of the best balances between sensitivity and specificity, with both measurements above 90%.

### ARIA Assistive Performance

Besides the sensitivity and specificity assessment, the ARIA system evaluation included 2 hybrid decision schemes, either assistive or a combination of human and AI. The experimental design was developed to reflect that in real-world applications, results of an automated system are reviewed and confirmed by health care professionals to choose the most adequate therapeutic protocol for each patient. In these assistive evaluations, we confirmed the existence of significant synergies derived from the interaction among the human and AI dyads.

The ARIA output’s influence on ophthalmologists’ overall precision depended on its format. A simplified output (ie, nonreferable or referable classification) resulted in the most successful sensitivity and specificity for ophthalmologists’ inputs. On the other hand, a more complex output (ie, with a confidence bar and attention map) partially improved ophthalmologists’ decisions, increasing their sensitivity but also increasing the incidence of false positive classifications.

These results are coherent with some of the ophthalmologists’ feedback submitted after the classification tasks, where some expressed that even when attention heatmaps were useful, the bar showing the confidence of the ARIA system was confusing.

### Limitations

Future pilot studies with a larger number of patients and ophthalmologists will be useful to confirm the ARIA system’s accuracy. Also, future studies might include direct ophthalmoscopy by retina specialists as the gold standard, in order to avoid errors related to image quality.

Additional experiments with alternative platform designs might be useful to generate a suitable screening tool that optimizes patient evaluations and referrals in 2 stages. In the first stage, an ARIA system might be useful to identify patients with a higher probability of developing DR. In the second stage, ophthalmologists would be able to evaluate the retinal images of high-risk patients, in combination with the ARIA system output, to make a first decision about the disease stage and treatment, sending referrals to retina specialists only for patients with an advanced disease.

### Conclusions

The results of this study demonstrate a substantial opportunity for Latin American countries such as Mexico toward developing efficient mass screening systems for early detection of diabetes-related blindness, considering the short supply of ophthalmologists in their public health care system.

The web-based platform developed for this study was designed for the implementation of the ARIA system as an automatic screening tool and as a telemedicine platform to confirm or reject the ARIA system’s output with assessment of an ophthalmologist or retina specialist. The platform was useful for this study and can be easily adapted for future studies that include the collection of additional information about other eye diseases detectable by image analysis (ie, glaucoma, age-related macular degeneration, or coat disease).

The conclusion of these results suggests the proposed ARIA system is valuable in an independent or assistive condition and can be useful to increase and improve DR diagnosis, as well as other ophthalmic diseases in the future. However, special attention to the design of an explanatory platform is required for successful implementation of the system.

## Appendix

Multimedia Appendix 1 Average number of observations according to the International Clinical Diabetic Retinopathy Severity Scales classification, for each set of 15 retina images in each treatment condition: solo, ARIA answer, and ARIA explanation.

Multimedia Appendix 2 CONSORT-EHEALTH checklist (V 1.6.1).

## Abbreviations

AI: artificial intelligence
ARIA: automated retinal image analysis
AUROC: area under the receiver operating characteristic curve
DL: deep learning
DR: diabetic retinopathy
ICDRSS: International Clinical Diabetic Retinopathy Severity Scales
NPDR: nonproliferative diabetic retinopathy

## References

[1] Zhang X, Saaddine JB, Chou C, Cotch MF, Cheng YJ, Geiss LS, Gregg EW, Albright AL, Klein BEK, Klein R (2010). Prevalence of diabetic retinopathy in the United States, 2005-2008. JAMA.

[2] SIGMA Type 2 Diabetes Consortium T, Williams Amy L, Jacobs Suzanne B R, Moreno-Macías Hortensia, Huerta-Chagoya Alicia, Churchhouse Claire, Márquez-Luna Carla, García-Ortíz Humberto, Gómez-Vázquez María José, Burtt Noël P, Aguilar-Salinas Carlos A, González-Villalpando Clicerio, Florez Jose C, Orozco Lorena, Haiman Christopher A, Tusié-Luna Teresa, Altshuler David (2014). Sequence variants in SLC16A11 are a common risk factor for type 2 diabetes in Mexico. Nature.

[3] Caballero AE (2011). Understanding the Hispanic/Latino patient. Am J Med.

[4] Secretaría de Salud (2016). 2016. Encuesta Nacional de Salud y Nutrición de Medio Camino 2016 (Ensanut MC 2016).

[5] Institute for Health Metrics and Evaluation 2017. Country profiles (Mexico).

[6] Barcelo Alberto, Arredondo A, Gordillo-Tobar A, Segovia J, Qiang A (2017). The cost of diabetes in Latin America and the Caribbean in 2015: Evidence for decision and policy makers. Journal of Global Health.

[7] International Diabetes Federation (2019). 2019. IDF Diabetes Atlas Ninth edition.

[8] Gómez Eduardo J (2020). Political party ambitions and type-2 diabetes policy in Brazil and Mexico. Health Econ Policy Law.

[9] Deshpande A, Harris-Hayes M, Schootman M (2008). Epidemiology of diabetes and diabetes-related complications. Phys Ther.

[10] Cheung N, Mitchell P, Wong TY (2010). Diabetic retinopathy. The Lancet.

[11] Secretaría de Salud (2011). Diagnóstico y Tratamiento de Retinopatía Diabética. Evidencias y Recomendaciones. Catálogo Maestro. Guías de Práctica Clínica.

[12] Jaime Claramunt L (2016). RETINOPATÍA DIABÉTICA DESDE LA PREVENCIÓN. INTEGRAR LA PESQUISA EN LOS CENTROS DE DIABETES. Revista Médica Clínica Las Condes.

[13] Barría VF, Martínez CF (2011). Clinical Practice Guide for Diabetic Retinopathy for Latin America for Ophthalmologists and Healthcare Professionals.

[14] Cavan D, Makaroff L, da Rocha Fernandes J, Sylvanowicz M, Ackland P, Conlon J, Chaney D, Malhi A, Barratt J (2017). The Diabetic Retinopathy Barometer Study: Global perspectives on access to and experiences of diabetic retinopathy screening and treatment. Diabetes Res Clin Pract.

[15] Jiménez Báez Mv, Márquez González H, Bárcenas Contreras R, Morales Montoya C, García Espinosa Lf (2015). EARLY DIAGNOSIS OF DIABETIC RETINOPATHY IN PRIMARY CARE. Colombia Médica.

[16] Carrillo-Alarcón L, Ávila-Pozos R, López LE, Cruz-Castillo R, Ocampo-Torres M, Alcalde-Rabanal J (2017). Projection of Diabetic Patients Retinopathy in Hidalgo State-México, through 2030. EC Ophthalmology.

[17] Thomas RL, Halim S, Gurudas S, Sivaprasad S, Owens D (2019). IDF Diabetes Atlas: A review of studies utilising retinal photography on the global prevalence of diabetes related retinopathy between 2015 and 2018. Diabetes Res Clin Pract.

[18] Hong H, Mújica Oscar J, Anaya J, Lansingh VC, López Ellery, Silva JC (2016). The Challenge of Universal Eye Health in Latin America: distributive inequality of ophthalmologists in 14 countries. BMJ Open.

[19] Martinez J, Hernandez-Bogantes E, Wu L (2011). Diabetic retinopathy screening using single-field digital fundus photography at a district level in Costa Rica: a pilot study. Int Ophthalmol.

[20] Salamanca O, Geary A, Suárez Nancy, Benavent S, Gonzalez M (2018). Implementation of a diabetic retinopathy referral network, Peru. Bull World Health Organ.

[21] Rachapelle S, Legood R, Alavi Y, Lindfield R, Sharma T, Kuper H, Polack S (2013). The cost-utility of telemedicine to screen for diabetic retinopathy in India. Ophthalmology.

[22] (2016). 2016. From $600 M to $6 Billion, Artificial Intelligence Systems Poised for Dramatic Market Expansion in Healthcare.

[23] He J, Baxter SL, Xu J, Xu J, Zhou X, Zhang K (2019). The practical implementation of artificial intelligence technologies in medicine. Nat Med.

[24] Arenas-Cavalli J, Ríos S, Pola M, Donoso R (2015). A Web-based Platform for Automated Diabetic Retinopathy Screening. Procedia Computer Science.

[25] Yang Y, Li T, Li W, Wu H, Fan W, Zhang W (2017). Lesion detection and Grading of Diabetic Retinopathy via Two-stages Deep Convolutional Neural Networks. Lecture Notes in Computer Science.

[26] Bhaskaranand M, Ramachandra C, Bhat S, Cuadros J, Nittala MG, Sadda S, Solanki K (2016). Automated Diabetic Retinopathy Screening and Monitoring Using Retinal Fundus Image Analysis. Journal of Diabetes Science and Technology.

[27] Gulshan V, Peng L, Coram M, Stumpe MC, Wu D, Narayanaswamy A, Venugopalan S, Widner K, Madams T, Cuadros J, Kim R, Raman R, Nelson PC, Mega JL, Webster DR (2016). Development and Validation of a Deep Learning Algorithm for Detection of Diabetic Retinopathy in Retinal Fundus Photographs. JAMA.

[28] Tufail A, Rudisill C, Egan C, Kapetanakis VV, Salas-Vega S, Owen CG, Lee A, Louw V, Anderson J, Liew G, Bolter L, Srinivas S, Nittala M, Sadda S, Taylor P, Rudnicka AR (2017). Automated Diabetic Retinopathy Image Assessment Software: Diagnostic Accuracy and Cost-Effectiveness Compared with Human Graders. Ophthalmology.

[29] Dutz M, Almeida R, Packard T (2018). The Jobs of Tomorrow: Technology, Productivity, and Prosperity in Latin America and the Caribbean.

[30] Chollet F, others (2015). Keras.

[31] Van RG, Drake JF (2001). Python Tutorial Release 2.

[32] Wilkinson C, Ferris FL, Klein RE, Lee PP, Agardh CD, Davis M, Dills D, Kampik A, Pararajasegaram R, Verdaguer JT, Global Diabetic Retinopathy Project Group (2003). Proposed international clinical diabetic retinopathy and diabetic macular edema disease severity scales. Ophthalmology.

[33] LeCun Y, Boser B, Denker JS, Henderson D, Howard RE, Hubbard W, Jackel LD (1989). Backpropagation Applied to Handwritten Zip Code Recognition. Neural Computation.

[34] Simonyan K, Zisserman A (2015). Very Deep Convolutional Networks for Large-Scale Image Recognition. ArXiv.

[35] Cuadros J, Bresnick G (2009). EyePACS: an adaptable telemedicine system for diabetic retinopathy screening. Journal of Diabetes Science and Technology.

[36] Voets M, Møllersen Kajsa, Bongo L (2019). Reproduction study using public data of: Development and validation of a deep learning algorithm for detection of diabetic retinopathy in retinal fundus photographs. PLoS One.

[37] Samek W, Montavon G, Binder A, La-puschkin S, Muller KR (2016). Interpreting the Predictions of Complex ML Models by Layer-wise Relevance Propagation. ArXiv.

[38] Bach S, Binder A, Montavon G, Klauschen F, Müller K, Samek W (2015). On Pixel-Wise Explanations for Non-Linear Classifier Decisions by Layer-Wise Relevance Propagation. PLoS One.

[39] Almaatouq A, Becker J, Houghton JP, Paton N, Watts DJ, Whiting ME (2021). Empirica: a virtual lab for high-throughput macro-level experiments. Behavior Research Methods.

[40] Saeedi P, Petersohn I, Salpea P, Malanda B, Karuranga S, Unwin N, Colagiuri S, Guariguata L, Motala AA, Ogurtsova K, Shaw JE, Bright D, Williams R, IDF Diabetes Atlas Committee (2019). Global and regional diabetes prevalence estimates for 2019 and projections for 2030 and 2045: Results from the International Diabetes Federation Diabetes Atlas, 9 edition. Diabetes Res Clin Pract.

[41] Barquera S, Campos-Nonato I, Aguilar-Salinas C, Lopez-Ridaura R, Arredondo A, Rivera-Dommarco J (2013). Diabetes in Mexico: cost and management of diabetes and its complications and challenges for health policy. Global Health.

[42] Raumviboonsuk P, Krause J, Chotcomwongse P, Sayres R, Raman R, Widner K, Campana Bilson J L, Phene Sonia, Hemarat Kornwipa, Tadarati Mongkol, Silpa-Archa Sukhum, Limwattanayingyong Jirawut, Rao Chetan, Kuruvilla Oscar, Jung Jesse, Tan Jeffrey, Orprayoon Surapong, Kangwanwongpaisan Chawawat, Sukumalpaiboon Ramase, Luengchaichawang Chainarong, Fuangkaew Jitumporn, Kongsap Pipat, Chualinpha Lamyong, Saree Sarawuth, Kawinpanitan Srirut, Mitvongsa Korntip, Lawanasakol Siriporn, Thepchatri Chaiyasit, Wongpichedchai Lalita, Corrado Greg S, Peng Lily, Webster Dale R (2019). Deep learning versus human graders for classifying diabetic retinopathy severity in a nationwide screening program. NPJ Digit Med.

[43] Beede E, Baylor E, Hersch F, Iurchenko A, Wilcox L, Ruamviboonsuk P, Vardoulakis L (2020). A Human-Centered Evaluation of a Deep Learning System Deployed in Clinics for the Detection of Diabetic Retinopathy.

[44] Nørgaard Mads Fonager, Grauslund J (2018). Automated Screening for Diabetic Retinopathy - A Systematic Review. Ophthalmic Res.

[45] Grzybowski A, Brona P, Lim G, Ruamviboonsuk P, Tan G, Abramoff M, Ting D (2020). Artificial intelligence for diabetic retinopathy screening: a review. Eye (Lond).

