# Supplementary material for: Screening Diabetic Retinopathy Using an Automated Retinal Image Analysis System in Independent and Assistive Use Cases in Mexico: Randomized Controlled Trial
Source: JMIR Form Res. 2021 Aug 26;5(8):e25290. doi: 10.2196/25290 (PMC8430849; doi:10.2196/25290)
Supplement: Multimedia Appendix 1 [file formative_v5i8e25290_app1.doc]

## Appendix

Table A1. Average number of observations according to the ICDRSS classification, for each set of 15 retina images in each treatment condition: *solo*, *ARIA answer* and *ARIA explanation*.

| **ICDRSS/**  **Condition** | **No apparent DR** | **Mild**  **DR** | **Moderate DR** | **Severe**  **DR** | **Proliferative DR** |
| --- | --- | --- | --- | --- | --- |
| solo | 7.5 | 0.0 | 5.3 | 1.5 | 0.7 |
| ARIA answer | 7.0 | 0.1 | 5.6 | 1.7 | 0.5 |
| ARIA explanation | 7.8 | 0.2 | 4.3 | 2.1 | 0.6 |

##


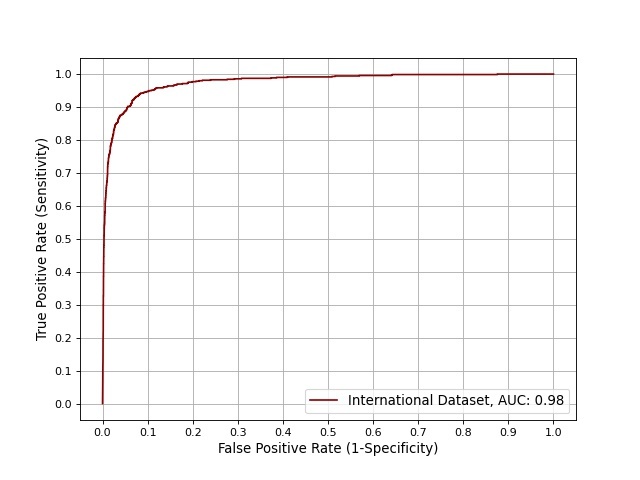
Figure A1. ROC curve of the ARIA system performance on the test set of international cases.
